# Using PVA and Attapulgite for the Stabilization of Clayey Soil

**DOI:** 10.3390/polym14214752

**Published:** 2022-11-05

**Authors:** Chengzong He, Guochang Hu, Hong Mei, Xiaoyong Zhu, Jian Xue, Jin Liu, Faming Zhang, Wenyue Che, Zhihao Chen, Zezhuo Song

**Affiliations:** 1 School of Earth Sciences and Engineering, Hohai University, Nanjing 210098, China; 2 Jiangsu Geology & Mineral Exploration Bureau, Nanjing 210002, China; 3 School of Engineering, Royal Melbourne Institute of Technology, Melbourne, VIC 3001, Australia

**Keywords:** polyvinyl acetate, attapulgite, cohesion and internal friction angle, water-holding capacity, scanning electron microscopy images

## Abstract

Considering that, in the context of the ecological restoration of a large number of exposed rock slopes, it is difficult for existing artificial soil to meet the requirements of mechanical properties and ecological construction at the same time, this paper investigates the stabilization benefits of polyvinyl acetate and attapulgite-treated clayey soil through a series of laboratory experiments. To study the effectiveness of polyvinyl acetate (PVA) and attapulgite as soil stabilizer, a triaxial strength test, an evaporation test and a vegetation growth test were carried out on improved soil with different amounts of PVA content (0, 1%, 2%, 3%, and 4%) and attapulgite replacement (0, 2%, 4%, 6%, and 8%). The results show that the single and composite materials of polyvinyl acetate and attapulgite can increase the peak deviator stress of the sample. The addition of polyvinyl acetate can improve the soil strength by increasing the cohesion of the sample; the addition of attapulgite improves the soil strength mainly by increasing the internal friction angle of the sample. The strength of the composite is greatly improved by increasing the cohesion and internal friction angle of the sample at the same time. The effect of adding materials increased significantly with increasing curing age. Moreover, polyvinyl acetate and attapulgite improve the soil water retention of the soil by improving the water-holding capacity, so that the soil can still ensure the good growth of vegetation under long-term drought conditions. The scanning electron microscopy (SEM) images indicated that the PVA and attapulgite of soil affect the strength characteristics of soil specimens by the reaction of PVA and water, which changes the structure of the soil and, by the interweaving of attapulgite soil particles, acts as the skeleton of the aggregate. Overall, PVA and attapulgite can effectively increase clayey soil stability by improving the cohesive force and internal friction angle of clayey soil.

## 1. Introduction

Due to the large number of human digging activities, the ecological balance has been seriously damaged, leaving a large number of exposed rocky slopes [1,2]. Exposed rocky slopes after wind and sun weathering, and slope bedrock weathering and unloading, are serious. Rock fragmentation means that dangerous rock pumice is scattered everywhere; under the action of rainfall and external forces, collapse, landslides and other hazards may occur, resulting in a loss of human property and even danger to human life [3,4]. Clay has a loose structure, strong water sensitivity and is softened by water, which causes a series of geotechnical engineering problems such as the uneven settlement of the foundation, foundation pit uplift and road instability. Moreover, the mechanical strength of clay has an impact on a series of engineering stability issues such as the retaining wall, slope stability and foundation-bearing capacity. As a traditional reinforcement material, cement is applied to the protection of bare rock slopes due to its low cost and effectiveness. The test results show that a small amount of cement is conducive to improving the strength and stability of a slope, but when its content is too high, it will lead to too-high compaction and pH value of the slope and affect the growth of vegetation [5]. For the ecological restoration of a large number of exposed rock slopes left behind by mining engineering and highway engineering, the external-soil spray seeding method is often used for bare rock slopes [6,7].

In order to make the external soil more effective for slope stability and ecological restoration, researchers have suggested innovative methods to improve the strength, permeability and water retention of soil through chemistry, physics or biology in recent years [8,9]. The use of polymers as chemical soil stabilization materials has attracted more and more attention because of its environmental protection characteristics [10,11]. The addition of polymer can significantly increase the compressive strength and cohesion of soil by filling, covering and wrapping the soil with its colloid; an investigation showed that the unconfined compressive strength and California bearing ratio (CBR) value of the composite soil increased by 52.8% and 72.8%, respectively, with the addition of 7% polymethacrylate (PMA) [12,13,14]. Louzada et al. [15] showed that the cohesion and internal friction angle of polyethylene terephthalate (PET) composite soil are improved. Liu et al. [16] found that the reduction in the volume change in expansive soil treated with polyvinyl acetate (PVA) was due to pore filling and particle wrapping caused by the polymer.

To further improve the stability of cohesive soil more significantly, the use of attapulgite to improve the soil has been studied by many researchers. Attapulgite is a clay mineral with a chain-layered structure and adsorption, colloid and catalytic properties [17,18,19]. In recent years, attapulgite has been gradually used in soil pollution remediation technology [20]. Elkady et al. [21] revealed that the shear strength of composite sand is improved by the addition of attapulgite. Chai et al. [22] found that the addition of attapulgite could improve the unconfined compressive strength of soil by reducing the actual moisture content of frozen soil.

Based on previous studies, PVA was used to improve the stabilization of the soil, including cohesion, water stability, erosion resistance and water retention. Moreover, the use of attapulgite was considered in order to improve the internal friction angle of the soil. In this study, PVA and attapulgite were used as environmental protection materials in order to achieve the stabilization of clay. The triaxial strength characteristics, water retention capacity and vegetation growth characteristics were assessed, combined with a micro mechanism analysis. The results of this study provide a basis for a proposed stabilization method with PVA and attapulgite for improving the properties of clayey soil in terms of the slope stability and foundation-bearing capacity.

## 2. Materials and Methods

### 2.1. Test Soil

**Figure 1:**
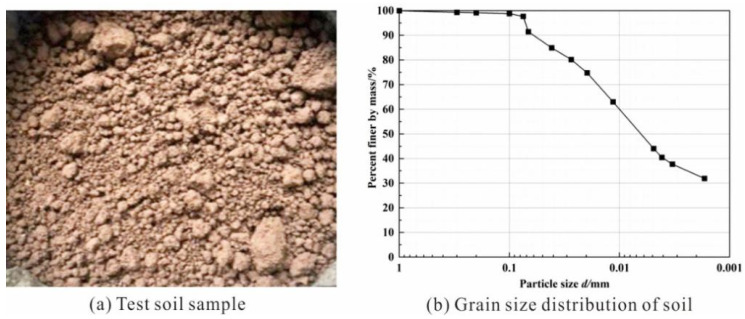
(**a**) Test soil sample; (**b**) grain size distribution of soil.

As shown in Figure 1a, high-quality soil was selected as the substrate component of the test soil, and the natural soil sample was relatively dry, yellow-brown, hard plastic, with uniform texture and no special odor, and contained a small amount of root impurities. Moreover, the test soil was collected in July 2021, and the GPS coordinates of its area are 118°45′24″ E, 31°54′34″ N. The area has limited vegetation on the surface and a large amount of exposed soil. There are traces of water erosion and soil erosion on the slope surface, which is prone to structural instability and deformation damage due to human activities and heavy rainfall. In total, 200 kg of the soil sample was collected and stored in a constant temperature environment of 25 °C. The grain size distribution of the soil is shown in Figure 1b. It has a specific gravity of 2.78, maximum dry density of 1.79 g/cm^3^, liquid limit of 35.8%, plastic limit of 17.7%, plasticity index of 18.1, and optimum moisture content of 19.1%.

### 2.2. Polyvinyl Acetate

Polyvinyl acetate (PVA, chemical formula [CH_3_COOCH_2_CH]_n_) is an ecological soil stabilizer, a milky white liquid composed of polyvinyl acetate with many long-chain macromolecules and polar carboxyl groups. The purity of the PVA, purchased from Guangzhou Qihua Chemical Co., Ltd. (Guangzhou, China), is > 99%. It is based on polyvinyl alcohol (chemical formula [C_2_H_4_O]_n_) as protective colloid, sodium dodecyl sulfate (SDS, chemical formula C_12_H_25_SO_4_Na) as emulsifier, and sodium persulfate (chemical formula Na_2_S_2_O_8_) as initiator, and acetic acid vinyl ester (VA, chemical formula C_4_H_6_O_2_) monomer emulsion is copolymerized in a polymerization kettle. 

### 2.3. Attapulgite

Attapulgite is a kind of hydrous magnesium-aluminum-silicate clay mineral with chain layer structure. The purity of the attapulgite, purchased from Lingshou Chenyang mineral products Co. (Shijiazhuang, China), Ltd., is > 99%. It is widely used for its special physical and chemical properties such as cation exchange, adsorption, colloid, catalytic and large specific surface area. It is used in petroleum, chemicals, building materials, paper, medicine, drilling and other industries. In each 2:1 unit structure layer, the corners of the tetrahedral wafers are reversed in direction at a certain distance, forming a chain-like structure between the chain-like structure and the layer-by-layer structure. The anisotropy of free oxygen atoms in the tetrahedron results in the fibrous extension of the attapulgite crystals. Due to its special crystal structure and physicochemical properties, attapulgite clay has good physical and chemical properties such as adsorption, colloid and catalysis.

### 2.4. Laboratory Tests

#### 2.4.1. Triaxial Test

The preparation of triaxial compression test samples is strictly in accordance with the layered compaction method in the standard for soil test methods (ASTM-D2850-15 Standard Test Method for Unconsolidated-Undrained Triaxial Compression Test on Cohesive Soils). The mixed soil samples are divided into three parts, and each layer is compacted 25 times to maintain the same layer height. The specimen has a height of 80 mm and a diameter of 39.1 mm. The prepared samples are sealed and wrapped with plastic film. After that, they are cured at room temperature (25 °C) for 14 days until the unconsolidated and undrained compression test is carried out.

In the triaxial compression test, the confining pressure is set to 50, 100, 150, and 200 kPa; the samples with the same content are parallel to each other under different confining pressures. The polyvinyl acetate content amount is set to 1%, 2%, 3%, and 4% of the clay mass. In order to avoid the influence of the attapulgite soil incorporation on changing the dry density of the specimen and resist the shear strength parameters, the scheme of attapulgite clay replacement and other quality clays was adopted, and the displacement amount of attapulgite soil was set to 2%, 4%, 6%, and 8% of the overall dry soil mass. Considering the influence of the later maintenance of the guest soil substrate and the root system after vegetation growth in actual construction, the change of shear strength parameters of the composite guest soil substrate in the early maintenance stage was mainly studied, and the maintenance age was set to 14 days. After studying the influence of polyvinyl acetate monomaterial on the shear strength parameters of the specimen, the two materials of polyvinyl acetate and attapulgite were mixed into the specimen in a 1:2 ratio according to the previously set parameters, and the performance of the two materials acting on the specimen when mixing was evaluated, and the maintenance age of the composite specimen was set to 14 days according to the analysis of the results of the single material test that had been carried out. After studying the effects of polyvinyl acetate and attapulgite soil single materials on the shear strength parameters of the specimen, the two materials were mixed into the specimen in a 1:2 ratio according to the previously set parameters, and the performance of the two materials acting on the specimen when mixing was evaluated, and the maintenance age of the composite material specimen was set to 14 days according to the analysis of the results of the single material test that had been carried out.

#### 2.4.2. Water Retention and Vegetation Growth Test

A transparent box of acrylic material of 20 cm × 10 cm × 10 cm was selected for the vegetation growth dishes. Ryegrass with high survival rate, strong adaptability, good greening effect and low cost was selected as the experimental grass species. The amount of polyvinyl acetate content/attapulgite soil replacement in the composite material was set to 0/0%, 1/2%, 2/4%, 3/6%, and 4/8%, and the detailed vegetation growth test scheme is shown in Table 1. We added water to the mixed material, loaded it into a vegetation growth dish and sprinkled 4 g of ryegrass seeds evenly. The quality of the specimen and the vegetation growth status were recorded daily.

## 3. Results and Discussion

### 3.1. Results of Triaxial Shear Test of Composite Specimens of Single PVA Material

As shown in Table 2, the variation of shear strength parameters of composite specimens of single polyvinyl acetate material under different content and curing age conditions was investigated by triaxial shear test. We controlled for the content of polyvinyl acetate—0, 1%, 2%, 3%, 4%—and curing age—14 days—and carried out comprehensive tests. 

**Figure 2:**
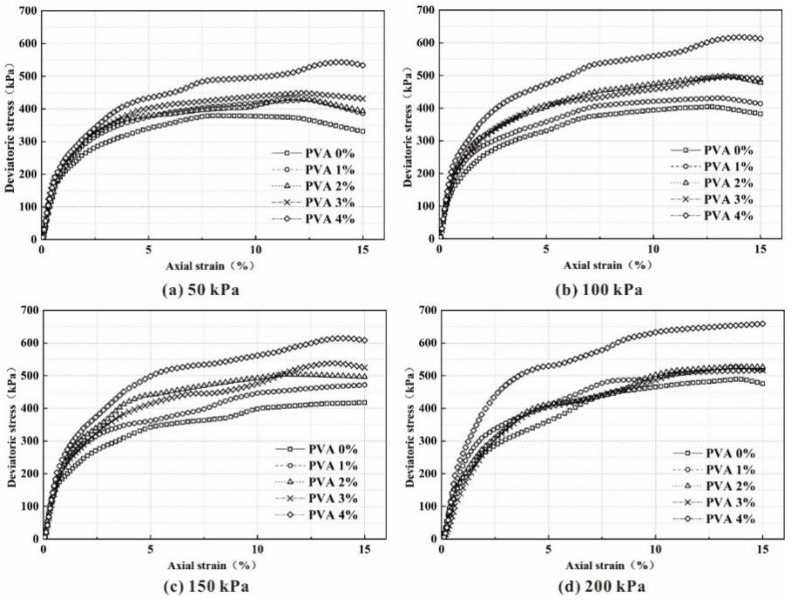
Deviator stress-strain curves with different amounts of polyvinyl acetate (PVA) under a confining stress of: (**a**) 50kPa; (**b**) 100 kPa; (**c**) 150 kPa; (**d**) 200 kPa.

In order to avoid the influence of too short a curing age on the action effect of polyvinyl acetate samples, first, we took each sample group at the curing age of 14 days as an example. The variation of shear strength parameters of triaxial shear test of different PVA content samples was analyzed. As shown in Figure 2, under each confining pressure, the slope of the elastic deformation stage of the partial stress–strain curve of the remolded sample increases with the increase in the amount of PVA, and the increasing trend of the slope gradually increases with the increase in confining pressure.

With the increase in the amount of PVA, the strain gradually delays when the peak value of the partial stress–strain curve appears, which improves the ability of the sample to resist elastic deformation. For example, when the confining pressure is 50 kPa, with increasing PVA content, the strains corresponding to the peak deviatoric stress are 8.25%, 12.25%, 11.75%, 12.25 and 14.00%. When the confining pressure is 100 kPa, with increasing polyvinyl acetate content, the strains corresponding to the peak deviatoric stress are 12.50%, 13.00%, 13.25%, 13.50% and 14.00%, respectively. With the continuous increase in confining pressure, the elastic limit and peak deviatoric stress of each group of samples increase, and their deviatoric stress–strain curves show strain hardening, which indicates that the addition of polyvinyl acetate can greatly improve the ability of the samples to resist elastic deformation.

**Figure 3:**
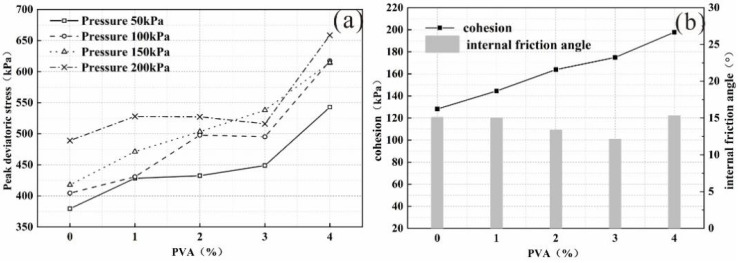
(**a**) The peak deviatoric stress; (**b**) cohesion and internal friction angle varied with PVA content.

Figure 3a shows the change curve of the peak deviatoric stress of each sample group when the curing age is 14 days under each confining pressure. The peak deviatoric stress of each sample group showed an obvious upward trend with increasing content of polyvinyl acetate. Taking the confining pressure of 50 kPa as an example, with the increase in the amount of polyvinyl acetate, the peak deviatoric stress of the samples was 379.52, 428.33, 432.53, 448.85 and 542.83 kPa, which is 13%, 14%, 18% and 43% higher than plain soil. Among them, the peak deviatoric stress of the sample group with a polyvinyl acetate content of 4% has the most significant increase, reaching 542.83, 617.34, 614.31, and 658.60 kPa under each confining pressure, which is higher than that of the plain soil sample, by 43%, 53%, 47% and 35%, respectively.

Variation curves of friction angle and cohesion in each sample group with a curing time of 14 days are plotted in Figure 3b. With the increase in the amount of polyvinyl acetate, the cohesion of the samples increased significantly, reaching 128.21, 144.48, 163.92, 174.89 and 197.74 kPa, which was 13 and 28% higher than that of plain soil, respectively, at 36 and 54%; the internal friction angle remained basically unchanged, ranging from 12 to 15°, which indicated that the incorporation of polyvinyl acetate mainly improved the soil strength by increasing the cohesion of the composite samples.

### 3.2. Analysis of Triaxial Shear Test Results of Attapulgite Composite Substrate

Attapulgite replacement amounts of 0, 2%, 4%, 6% and 8% and curing age of 14 days were applied to a total of 25 sample groups. The triaxial compression test results of each sample group are shown in Table 3.

**Figure 4:**
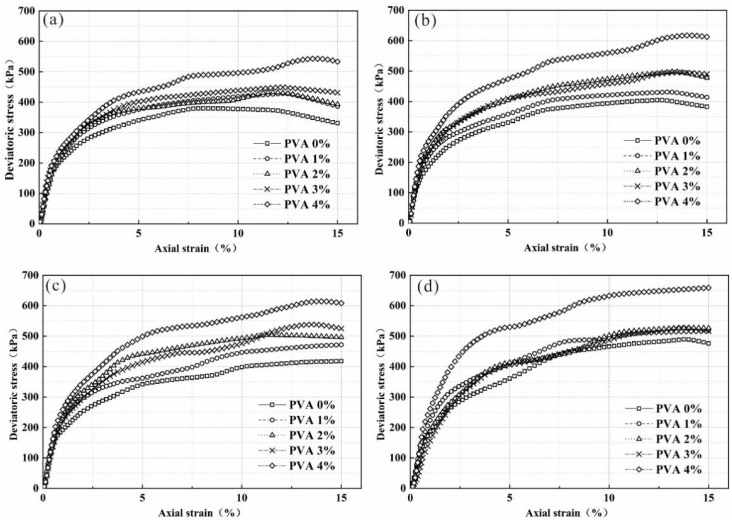
Deviator stress–strain curve with a curing time of 14 days under a confining stress of: (**a**) 50kPa; (**b**) 100 kPa; (**c**) 150 kPa; (**d**) 200 kPa.

Taking the shear strength parameters of the samples with a curing age of 14 days as an example, the shear strength parameters with the replacement amount of attapulgite when the curing age of the samples is higher were analyzed. As shown in Figure 4, under each confining pressure, with increasing attapulgite replacement amount, the slope and deviatoric stress level of the elastic stage of the sample increase as a whole, and with growing confining pressure, the deviatoric stress level of the sample increases. The increase is gradual.

**Figure 5:**
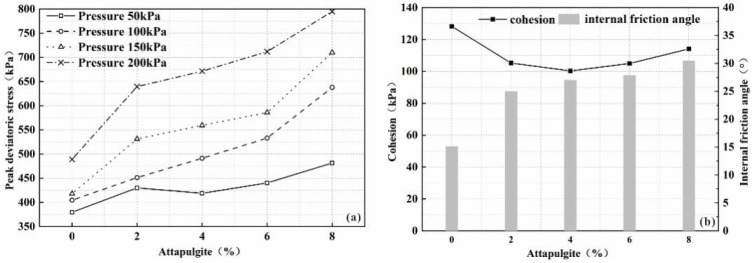
(**a**) The peak deviatoric stress; (**b**) cohesion and internal friction angle varied with attapulgite replacement.

Figure 5a shows the peak deviatoric stress curve with a curing time of 14 days. Under each confining pressure, with increasing amount of attapulgite soil replacement, the peak deviatoric stress of the sample shows an overall upward trend. At the same time, the peak deviator stress of the sample also increases with increasing confining pressure, which again shows that the addition of attapulgite improves the strength characteristics of the sample.

Figure 5b shows the curves of the internal friction angle and cohesion of each sample group with a curing age of 14 days. With increasing attapulgite content, the cohesion of the samples is 100.29, 104.92 and 114.09 kPa, and the internal friction angles are 15.10°, 24.99°, 27.00°, 27.87° and 30.43°, indicating that the attapulgite soil mainly improves the strength of the soil by increasing the internal friction angle of the sample.

This is directly related to the shape and structure of attapulgite. The fibrous particles of attapulgite give it a large specific surface area and show strong adsorption, and the clay particles adhere to the particle surface of attapulgite to form aggregates, the agglomerate–agglomerate combination leads to a denser occlusal state, which increases the occlusal friction force when the soil is damaged, thereby increasing the internal friction angle of the soil. On the other hand, compared with clayey soil particles, attapulgite particles have stronger adsorption to water, and any water in the clayey soil tends to be more easily adsorbed in the presence of attapulgite particles.

### 3.3. Analysis of Triaxial Compression Test Results of Polymer and Attapulgite Composite Substrates

The variation law of shear strength parameters of polyvinyl acetate and attapulgite composite substrates was analyzed by triaxial shear test and compared with that of single-material composite substrates. The parameters of the triaxial compression test results of composite samples with a curing time of 14 days are shown in Table 4.

**Figure 6:**
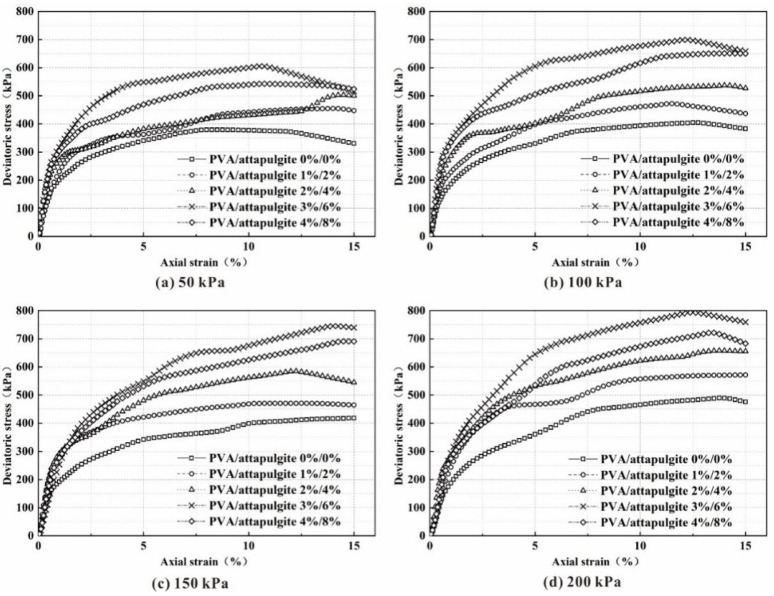
The deviatoric stress–strain curve of reinforced specimens under a confining pressure of: (**a**) 50 kPa; (**b**) 100 kPa; (**c**) 150 kPa; (**d**) 200 kPa.

The deviatoric stress–strain curve of the sample is shown in Figure 6. Under each confining pressure, the deviatoric stress of the sample shows a trend of first increasing and then decreasing with increasing composite material content, with 3%/6% as the peak value. This shows that when the curing age is 14 days, there is a threshold for the effect of composite materials on soil strength, and further increasing the content of composite materials will reduce the soil strength.

In the composite sample, the elastic modulus of the sample is affected by the confining pressure and gradually increases with increasing composite material content, but compared with the plain soil sample, the incorporation of the composite material can improve the elastic modulus of the sample.

**Figure 7:**
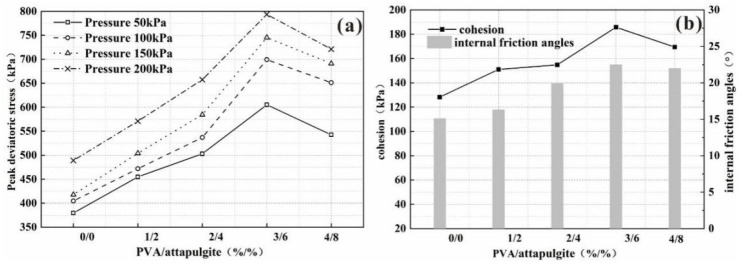
(**a**) The peak deviator stress curve; (**b**) cohesion and internal friction angle curve with different PVA content and attapulgite replacement.

The change curve of peak deviatoric stress and the change curve of cohesion and internal friction angle are shown in Figure 7a and Figure 7b, respectively. Under each confining pressure, the peak deviatoric stress, cohesion and internal friction angle of the samples increased first and then decreased with increasing composite content, and the composite content corresponding to the peak value was 3%/6%. The internal friction angles of the samples are 15.10°, 16.29°, 19.91°, 22.49° and 21.97°, and the cohesion of the samples is 128.21, 151.01, 154.80, 185.75 and 169.43 kPa, respectively.

### 3.4. Comparison of Triaxial Compression Test Results between Composite and Single-Material Composite Substrate

**Figure 8:**
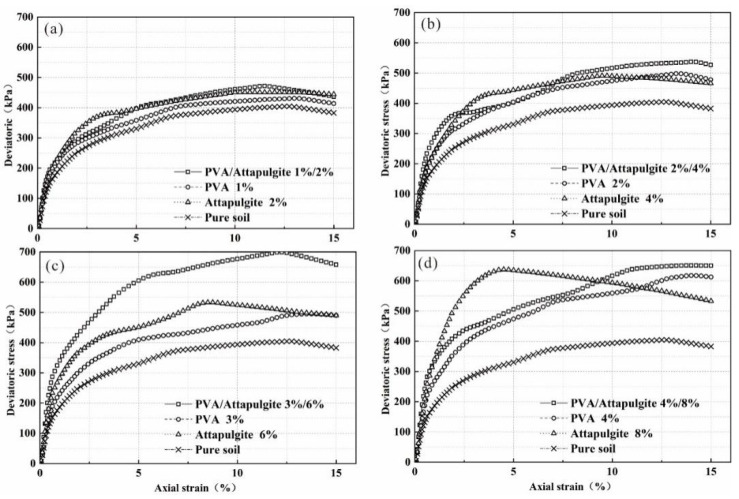
Deviatoric stress–strain curve under a confining stress of 100 kPa: (**a**) PVA content: 1%, attapulgite: 2%; (**b**) PVA content: 2%, attapulgite: 4%; (**c**) PVA content: 3%, attapulgite: 6%; (**d**) PVA content: 4%, attapulgite: 8%.

Taking the sample with a confining pressure of 100 kPa as an example, the triaxial compression test results of single material and composite material of polyvinyl acetate and attapulgite with curing age of 14 days are compared. The deviatoric stress–strain curve is shown in Figure 8. It can be seen that with increasing material content, the failure strain of the polyvinyl acetate composite sample gradually increases and that of attapulgite composite sample gradually decreases. The failure strain of composite samples is between two single-material composite samples.

Taking Figure 8c as an example, the failure strains of attapulgite composite sample, composite sample, plain soil sample and polyvinyl acetate composite sample are 8.5, 12.25, 12.5 and 13.5%, respectively, which shows that the addition of polyvinyl acetate in the composite can effectively reduce the improvement effect of attapulgite on the brittleness of the sample. The partial stress-strain curve of the composite specimen tends towards the plastic failure type of polyvinyl acetate composite specimen.

**Figure 9:**
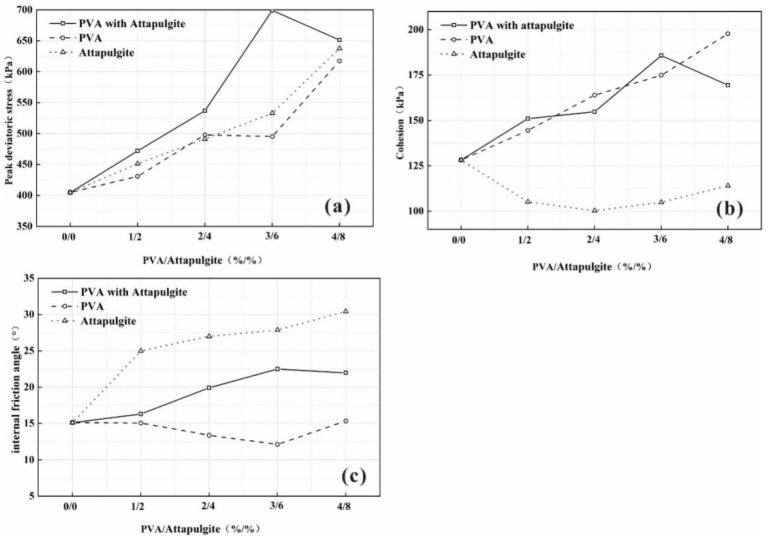
(**a**) The peak deviator stress curve, (**b**) cohesion curve and (**c**) internal friction angle with different amounts of PVA and attapulgite replacement.

The variation curve of the peak deviatoric stress of the specimen with a confining pressure of 100 kPa is shown in Figure 9a. Compared with the single-material composite specimen, as the material content increases, the peak deviatoric stress of the composite material specimen increases. Different from the variation trend of the peak deviatoric stress of the single-material composite sample, within the set variable range, the peak deviatoric stress of the composite sample appears to peak at a composite content of 3%/6%.

The variation curves of cohesion and internal friction angle of the sample are shown in Figure 9b and Figure 9c, respectively. Through comparison with the triaxial compression test results of single-material composite sample, it can be seen that polyvinyl acetate mainly increases the soil strength by improving the cohesion of the sample, and attapulgite mainly increases the soil strength by increasing the internal friction angle of the sample. The composite material can improve the soil strength by increasing the cohesion and internal friction angle of the sample at the same time.

### 3.5. The Effect of Water Retention and Plant Growth on Soil

**Figure 10:**
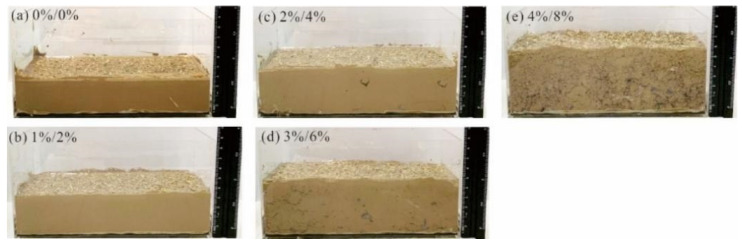
Initial state of the composite sample: (**a**) PVA content: 0%, attapulgite: 0%; (**b**) PVA content: 1%, attapulgite: 2%; (**c**) PVA content: 2%, attapulgite: 4%; (**d**) PVA content: 3%, attapulgite: 6%; (**e**) PVA content: 4%, attapulgite: 8%.

Figure 10 shows the initial state of the composite sample. It can be seen that with increasing composite material content, the macroporosity of the sample increases significantly, and the sample volume gradually increases. The corresponding sample heights are 3.7, 4.4, 4.8, 5.9 and 6.9 cm, which were increased by 19%, 30%, 59% and 86%, compared with plain soil. At the same time, with increasing composite material content, the water-holding capacity of the samples gradually increased, and the corresponding water-holding capacities are 402, 503, 603, 771 and 938 g, respectively, which increased by 25%, 50%, 92% and 133% compared with the plain soil. 

This shows that the incorporation of composite materials increases the aggregate structure of the clay, which increases the total porosity and effective porosity of the clay, and significantly improves the water-holding capacity of the composite samples.

**Figure 11:**
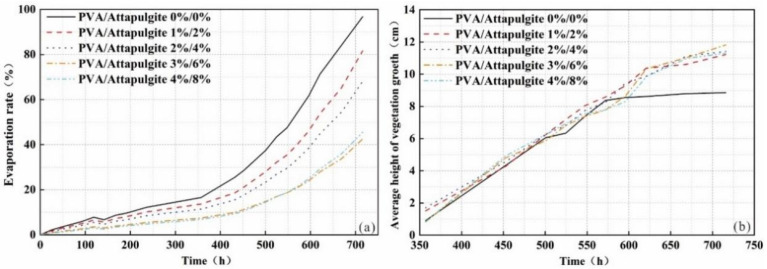
(**a**) The evaporation rate and (**b**) average height with different PVA content and attapulgite replacement.

The evaporation rate–time curve of each group of samples is shown in Figure 11a. The evaporation rate of each sample increases linearly from 0 to 358 h, and the evaporation rate is almost unchanged. After 358 h, the vegetation of each sample begins to germinate, and the resulting transpiration causes a gradual increase in the evaporation rate of the sample.

With increasing composite material content, the evaporation curve of the sample gradually decreases, and the corresponding average evaporation rates are 0.14, 0.11, 0.10, 0.06 and 0.06 %/h. This indicates that the incorporation of composites improves the water retention of the samples.

The average height–time variation curve of the vegetation growth of the sample is shown in Figure 11b. All samples began to germinate at about 357 h. The average height of vegetation of each sample increased linearly and rapidly at 357~572 h, and the difference of average height of vegetation of each sample was not obvious. After 572 h, the growth rate of average height of vegetation of the composite sample decreased relatively, and the vegetation of plain soil sample basically stopped growing. At 716 h, with increasing composite content, the average height of vegetation of the samples reached 8.8, 11.2, 11.4, 11.8 and 11.3 cm.

**Figure 12:**
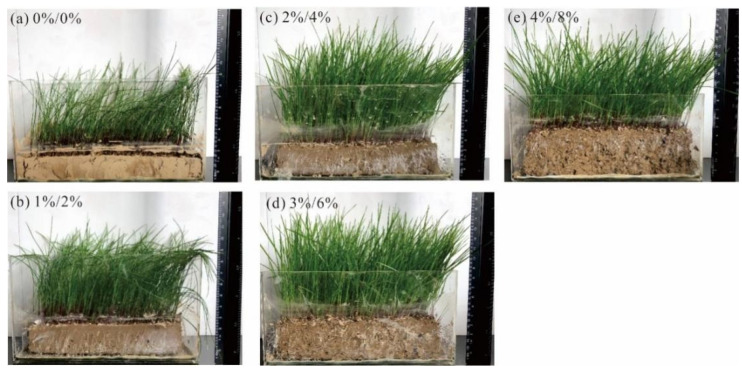
Vegetation growth state in 716 h: (**a**) PVA content: 0%, attapulgite: 0%; (**b**) PVA content: 1%, attapulgite: 2%; (**c**) PVA content: 2%, attapulgite: 2%; (**d**) PVA content: 3%, attapulgite: 6%; (**e**) PVA content: 4%, attapulgite: 8%.

Figure 12 shows the vegetation growth state of each sample at 716 h. The vegetation leaves of the soil samples are shriveled and bent, and many large cracks appear in the samples due to cracking. With increasing content of composite materials, the vegetation leaves of the composite sample are fuller, straight, and without cracks, and the sample shrinks inward as a whole. This shows that the addition of composite materials will not have an adverse impact on the growth of vegetation and can improve the water-holding capacity and water retention of soil, so that the soil can still ensure the better growth of plants under long-term drought conditions, and improve soil fertility.

### 3.6. The Effect of PVA and Attapulgite on the Mechanical Properties of Soil

**Figure 13:**
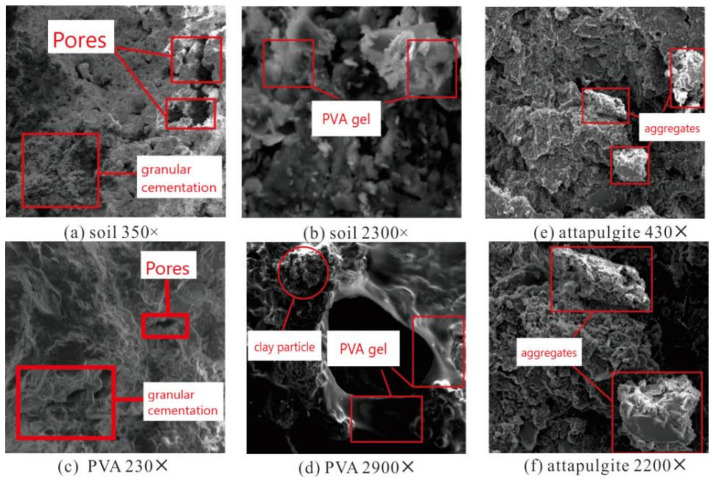
SEM image: (**a**) 350 times magnification of soil; (**b**) 2300 times magnification of soil; (**c**) 230 times magnification of PVA composite sample; (**d**) 2900 times magnification of PVA composite sample; (**e**) 430 times magnification of attapulgite composite sample; (**f**) 2200 times magnification of attapulgite composite sample.

The SEM image of the test soil sample is shown in Figure 13a,b. Under the magnification of 350× in Figure 13a, the test soil sample has distinct particles, the soil particle size is relatively average, the particles are cemented together, many small pores are evenly distributed, there are a few large pores and the overall structure is relatively loose. Under the magnification of 2300× in Figure 13b, the cementation formed by the soil particles through the soil particles is relatively uniform as a whole, with many pores interspersed between them.

An SEM image of the composite sample with a polyvinyl acetate content of 3% is shown in Figure 13c,d. It can be clearly seen that the elastic gel formed by the reaction of polyvinyl acetate and water effectively changes the structure of the clay, mainly due to its own viscosity and tensile strength. The effect of polyvinyl acetate gel on the clay structure can be summarized as covering, wrapping and filling. The polyvinyl acetate gel covers the surface of the clay particles and wraps them, and the formed agglomerates increase the integrity of the clay structure; at the same time, the cohesiveness of the gel increases the force between the clay particles. The clay pores are increased, the effective contact area between sand particles is increased, and the cohesion of the polyvinyl acetate composite sample is significantly improved.

It is clear from Figure 13e,f that the size of aggregates in attapulgite composite samples is different, and many aggregates have flake and long strip shapes, which may be related to the chain lamellar crystal structure of attapulgite. The formation of aggregates is mainly determined by the adsorption of attapulgite. According to different causes, the adsorption of attapulgite is mainly divided into three categories: physical adsorption, chemical adsorption and ion-exchange adsorption. Due to the large specific surface area of attapulgite, physical adsorption is the most obvious. The grid structure formed by the interweaving of attapulgite soil particles acts as the skeleton of the aggregate, which absorbs and adheres to the surrounding soil particles and water, increasing the integrity of the aggregate. Compared with the uniform cementation between soil particles and soil particles in plain soil samples, the cementation between aggregates and aggregates in composite samples has higher sliding friction and occlusal friction. Therefore, the internal friction angle of attapulgite composite samples is significantly improved.

According to the SEM image analysis, the elastic gel formed by the reaction of polyvinyl acetate and water effectively changes the structure of the clay, increases the clay pores, increases the effective contact area between sand particles, and significantly improves the cohesiveness of the sand. The microstructure verified that the improvement in the triaxial strength test of cohesive soil after PVA mainly occurred by increasing the cohesion between particles. In addition, the replacement of attapulgite makes the cohesive soil aggregate and the cementation between aggregates have a higher sliding friction and bite friction, which also verifies that the triaxial strength of the modified clayey soil is improved mainly by increasing the internal friction angle between the soil particles.

**Figure 14:**
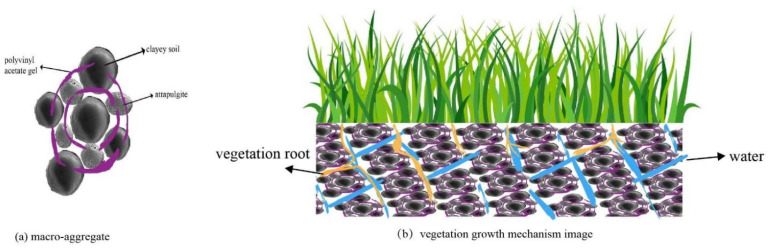
(**a**) Modified clayey soil with PVA and attapulgite; (**b**) vegetation growth mechanism image of modified clayey soil.

In Figure 14a, the particle gaps in the modified clayey soil are fully filled by attapulgite and wrapped in polymer film, which helps to improve the stability of the modified clayey soil under a mechanical load or hydraulic action. The formation of aggregates not only results in cohesion, but also their strong attraction to one another, thus enhancing the stability of the clayey soil. In Figure 14b, the structure of the clayey soil improved by PVA and attapulgite becomes dense, the physical strength is enhanced and the stability is significantly improved. The soil strength increases and is not easily compromised, and the voids between particles become smaller. Therefore, the evaporation rate of water is reduced and the water is fully preserved. Overall, the vegetation growth effect of the modified soil is improved.

This experimental investigation into the PVA- and attapulgite-based stabilization of clayey soil reveals that PVA and attapulgite effectively improved the mechanical and water retention properties. Moreover, the addition of PVA and attapulgite can also benefit the growth of plants.

The main mechanism of PVA in stabilizing clayey soil lies in the fact that the polar carboxyl long-chain macromolecules on its surface react with hydroxyl or alkali metal ions in the soil to increase the adhesion between soil particles [23,24]. In addition, the continuous elastic membrane formed in the clayey soil matrix after curing with polymer solution can act as a bridge connecting the soil [25,26]. These characteristics are shown in Figure 13c,d. The addition of PVA forms colloids with soil particles and fills the voids between soil particles, which improves the cohesion of the sample.

Attapulgite mainly improves the soil by filling the gaps between the soil particles and increasing the internal friction of the soil. Zhang et al. [27] revealed that the mechanism of attapulgite-modified loess is that attapulgite fills the pores between loess particles and forms a sheet-like integral structure with loess, thus reducing the permeability coefficient. Furthermore, Chen et al. [28] revealed that attapulgite has a special crystal structure and alternating properties, which provide it with excellent ion exchange and surface adsorption properties, which can effectively improve the physical properties of soil. In Figure 13e,f, we found that the addition of attapulgite can act as the skeleton of the aggregate, which absorbs and adheres to the surrounding soil particles and water, increasing the integrity of the aggregate.

Soil water-holding capacity is one of the most important factors affecting vegetation growth. Zhou et al. [29] reported that the special molecular structure of polyvinyl alcohol enables it to be combined with water molecules, so as to absorb and store a large amount of water in the long polymer chain. On the other hand, Yang et al. [17] revealed that when attapulgite was applied to soil, the soil moisture evaporation and cracking behavior changed significantly. In particular, the effect of adding 3% attapulgite was the best. This is because the spatial structure and particles of cohesive soil change significantly, which makes the loess particles more compact. Moreover, the addition of PVA also significantly improved the porosity and reduced the heat exchange between the air and soil, thereby improving the plant growth conditions [30,31]. As shown in Figure 11 and Figure 12, the evaporation rate of clayey soil gradually decreases with the increase in the PVA content, as well as with the increase in the attapulgite content. When the content of PVA is 3% and that of attapulgite is 6%, the water retention of the sample is the best and, at the same time, the vegetation growth is also the best.

In order to understand why PVA and attapulgite can overcome the limitations of clayey soil, a series of comparisons were made with the results of other authors. PVA’s effect on soil structure—in terms of the covering, wrapping, filling, and forming of agglomerates—increases the integrity of clayey soil. The grid structure formed by the interweaving of attapulgite particles acts as the skeleton of the aggregate, which absorbs and adheres to the surrounding soil particles and water, increasing the integrity of the aggregate. While Ilhan Chang et al. [32] revealed that the strengthening effect of Xanthan gum increases in the presence of grained soil, this is most likely the result of Xanthan gum’s interactions with the charged surfaces of clayey soils. The direct interaction between Xanthan gum and clayey soil forms firm biopolymer–soil matrices, which act as cementeous binders between sandy particles. Al Swaidani Aref et al. [33] analyzed natural pozzolana and lime when added to soil and the results of the scanning electron microscopy (SEM) and energy-dispersive X-ray spectroscopy (EDX) showed significant changes in the microstructure of the treated clayey soil. Better flocculation of the clayey particles and the further formation of cementing materials in the natural pozzolanalime-treated clayey soil were clearly observed. Both PVA and attapulgite can fill the intervals within clayey soil, and lower permeability can be manufactured by the lower void ratio [11].

## 4. Conclusions

To study the effectiveness of PVA and attapulgite as soil stabilizers, a triaxial strength test, an evaporation test and a vegetation growth test were conducted on improved soil with different amounts of PVA and attapulgite replacements. The conclusions can be summarized as follows:

(1) The addition of polyvinyl acetate can greatly improve the strain capacity of the sample and resist elastic deformation. With increasing PVA content, the peak partial stress and cohesion of the sample are significant, and the maximum cohesion reaches 197.74 kPa, whereas the internal friction angle of the sample remains basically unchanged, which indicates that the improvement of soil strength by polyvinyl acetate is mainly achieved by improving the cohesion of the sample. With the increasing content of attapulgite, the peak partial stress and internal friction angle of the sample increase significantly, the maximum internal friction angle reaches 30.43°, and the cohesion decreases, which indicates that attapulgite mainly improves the strength of the soil by increasing the internal friction angle of the soil.
(2) Under the condition of curing age of 14 days, with increasing composite material content, the peak deviatoric stress, cohesion and internal friction angle of the sample first increased and then decreased, and the composite material content corresponding to the peak point was 3%/6%. Compared with the single-material composite samples with the corresponding content, the composite materials can improve the soil strength by increasing the cohesion and internal friction angle of the samples at the same time.
(3) With increasing composite material content, the water-holding capacity of the samples increased gradually, increasing by 25, 50, 92 and 133%, compared with the plain soil samples, and the average evaporation rate was higher than that of the plain soil samples, which was reduced by 19, 39, 85 and 129%, respectively. Compared with the plain soil samples, the vegetation leaves of the composite samples are fuller and taller, indicating that the incorporation of the composite material improves soil fertility, so that it can still ensure better plant growth under long-term drought conditions.
(4) The effect of PVA gel on the soil structure can be summarized as covering, wrapping and filling, and formed agglomerates increase the integrity of the clay structure and soil structure. The adsorption of attapulgite is mainly divided into three categories: physical adsorption, chemical adsorption and ion-exchange adsorption. The grid structure formed by the interweaving of attapulgite soil particles acts as the skeleton of the aggregate, which absorbs and adheres to the surrounding soil particles and water, increasing the integrity of the aggregate. Therefore, the cohesion of the polyvinyl acetate composite sample is significantly improved, and the internal friction angle of attapulgite composite samples is significantly improved.

## Figures and Tables

**Table 1:** Vegetation growth test protocol.

Specimen Number	Clay Quality (g)	Polyvinyl Acetate (PVA) Content (g)	Attapulgite Replacement (g)	Water (g)
S1	670.0	0	0	402
S2	656.6	6.7	13.4	503
S3	643.2	13.4	26.8	603
S4	629.8	20.1	40.2	771
S5	616.4	26.8	53.6	938

**Table 2:** Results of triaxial shear test of polyvinyl acetate specimens.

Curing Time (d)	PVA (%)	Peak Bias Stress of the Triaxial Test Under Each Confining Pressure (kPa)				Internal Friction Angle (°)		Cohesion (kPa)	Correlation Coefficient (R^2^)
		50 kPa	100 kPa	150 kPa	200 kPa				
14	0.00	379.52	404.58	417.75	489.12	15.10	128.21		0.8821
	1.00	428.33	430.74	471.29	527.92	15.05	144.48		0.8835
	2.00	432.53	497.86	503.39	527.27	13.38	163.92		0.8510
	3.00	448.85	495.14	537.97	546.04	12.13	174.89		0.9310
	4.00	542.83	617.34	614.31	658.60	15.33	197.74		0.8547

**Table 3:** Parameters of triaxial shear test results of attapulgite composite samples.

Curing Time (d)	Attapulgite Replacement (%)	Triaxial Peak Deviatoric Stress (kPa)				Internal Friction Angle (°)	Cohesion (kPa)	R^2^
		50 kPa	100 kPa	150 kPa	200 kPa			
14	0.00	379.52	404.58	417.75	489.12	15.10	128.21	0.8821
	2.00	429.97	451.27	531.18	639.52	24.99	105.20	0.9283
	4.00	418.78	491.27	559.32	671.39	27.00	100.29	0.9853
	6.00	440.27	532.92	585.82	711.90	27.87	104.92	0.9762
	8.00	481.54	637.66	710.00	794.92	30.43	114.09	0.9673

**Table 4:** Triaxial shear test result parameters of composite specimens.

Curing Time (d)	Composite Content (%/%)	Triaxial Peak Deviatoric Stress (kPa)				Internal Friction Angle (°)	Cohesion (kPa)	R^2^
		50 kPa	100 kPa	150 kPa	200 kPa			
14	0/0	379.52	404.58	417.75	489.12	15.10	128.21	0.8821
	1/2	454.82	471.88	504.09	570.98	16.29	151.01	0.9189
	2/4	503.00	536.90	584.51	657.53	19.91	154.80	0.9711
	3/6	605.12	699.18	745.18	793.61	22.49	185.75	0.9665
	4/8	542.52	651.15	691.09	721.14	21.97	169.43	0.9062

## Data Availability

The data presented in this study are available on request from the corresponding author.
